# Engineering of new graphene-based materials as potential materials to assist near-infrared photothermal therapy cancer treatment

**DOI:** 10.1016/j.heliyon.2020.e04131

**Published:** 2020-06-10

**Authors:** Faith Cheung

**Affiliations:** Richard Montgomery High School, 250 Richard Montgomery Drive, Rockville, MD 20852, United States

**Keywords:** Biomedical engineering, Materials chemistry, Materials science, Oncology, Physical chemistry, Photothermal therapy, Band gap, Graphene, First principle calculation

## Abstract

Photothermal therapy is an emerging method of cancer treatment in which tumors are ablated by heating agents using near-infrared light (700–1000 nm). A semiconductor with a band gap between 0.3–0.7 eV would therefore efficiently emit near-infrared light. The new “magic” material graphene has a band gap of zero, which is advantageous with regard to designing a new material with a suitable band gap for the emission of near infrared light. In our investigations, using the first principles density functional theory calculation method, we aimed to and successfully designed graphene-based materials with a direct band gap of 0.68 eV. They have the potential to be optimal and efficient near-infrared light sources, due to their narrow yet fitting band gap. The present results open up a new avenue for the application of graphene-based materials to assist in photothermal therapy.

## 1 Introduction

Chemotherapy, surgery, radiotherapy and even chemo-radiotherapy cancer treatments have been in use for a long time, and yet cancer metastasis remains one of the most critical challenges in the medical field, as well as one of the primary causes of cancer mortality [1, 2]. The metastatic process begins with the detachment of cancer cells from the primary tumor, followed by the infiltration of the lymphatic and circulatory systems and then multiplication and reproduction in various organs, leading to unstoppable growth of malignant tumors [3, 4]. Various methods for the treatment of cancer metastases have been explored in the past 200 years. One of them is surgery, which is usually highly invasive [5] and results in considerable long-lasting postoperative pain as well as a high risk of infection. For this reason, many cancer patients choose not to partake in this treatment, thereby facilitating the spread of cancer throughout the body [6]. Another option is represented by minimally invasive treat-ments, such as radiotherapy and chemotherapy. However, these two methods often result in the damage to or the destruction of healthy tissues in the vicinity of the tumor being ablated [7]. Unfortunately, a minimally invasive yet reliable and effective treatment has not yet been established. Therefore, although our understanding of cancer metastasis is growing, we are still a long way from committing to a reliable and efficient cure [8, 9] in this regard.

Photothermal therapy (PTT) is less damaging among other cancer treatment methods largely due to its creative use of heat [10]. This approach dates back to the 19th century, when doctors noticed tumor regression in patients with fever [11]. The approach uses near-infrared light photo-absorbers as photosensitizing agents to generate heat in near-IR-irradiated cancer cells, resulting in the ablation and death of those cells [12, 13]. This simple method can be used to combat initial-stage tumors and suppress their spread whilst largely leaving nearby tissues unaffected [14]. Moreover, the combination of photothermal therapy, along with other existing methods, such as chemo and radiotherapy, can also be used to treat metastatic tumors, guaranteeing the effectiveness of treatment [15]. The generation of photothermal energy depends on the conversion of near-infrared light into heat by nanoscale materials [14]. In particular, recent applications have employed photothermal agents such as gold nanoparticles and carbon nanotubes [12]. These nanomaterials play a key role in fields such as technology and medicine [16].

Graphene has various potential applications in the biomedical field [17]. In recent studies, graphene has shown high effectiveness in *in vitro* drug delivery as well as *in vivo* cancer treatment using a near-infrared laser source [18]. Moreover, graphene oxide can achieve better cancer cell targeting and photoablation performances if administered at a low dosage [19, 20].

This indicates that graphene oxide is an effective photothermal agent with an absorbance ability which is comparable to that of gold nanoparticles and carbon nanotubes [21]. However, graphene oxide's band gap is difficult to manage because the concentration and position of oxygen is difficult to control, making the efficiency of near-infrared light hard to control as well. In order to make graphene a more efficient photothermal agent, it is necessary to design new graphene-based materials with the band gap of 0.3–0.7 eV.

In this research, we used first-principles calculations to tune the band gap of graphene-based materials using the method of doping with different elements. In order to achieve the band gap within the aforementioned range to emit near infrared light [22], we deposited various dopants on top of a graphene sheet, interstitially doping graphene as well as replacing the carbon atoms in a graphene hexagonal with dopant atoms, substitutionally doping graphene and performing convergence, relaxation, and band structure calculations. In this way, we can identify efficient graphene-based materials that can be applied in photothermal therapy [23].

Our first-principles calculations, based on the density functional theory (DFT) method, are discussed in Section 2, while the results are reported in Section 3 and discussed in Section 4. Finally, Section 5 includes an analysis of the impact of these findings, along with some concluding remarks.

## 2 Method

We performed first-principles calculations based on the DFT [24, 25, 26, 27] method, using the Perdew-Burke-Ernzerhof (PBE) [28] exchange correlation (XC) [29] functional within the gener-alized gradient approximation (GGA). Projector augmented wave (PAW) [30] pseudopotentials were generated from atomic data using the AtomPAW code [31].

The ABINIT [32] package was used to perform all DFT calculations.

The convergence criterion for total energy calculations in the self-consistent field (SCF) iterations were set to 10^−10^ Hartee/Bohr. Structural optimization was performed using.

Broyden-Fletcher-Goldfarb-Shanno method (BFGS) [33] and the convergence criterion for total forces in the SCF iterations were set to 10^−6^ Hartree/Bohr.

First of all, we needed to perform convergence for kinetic energy cutoff, k-point mesh and vacuum height. The convergence criterion for the total energy were less than 0.0001 Hartree for two consecutive SCF cycles. In order to deposit impurities on top of a graphene sheet, we used the above mentioned converged kinetic energy cutoff, k-point mesh and vacuum height values to perform optimization. The atom position and the size of the unit cell were relaxed until the equilibrium state was reached. In the next step, we used all the above-mentioned converged parameters and optimized the atom position and unit cell to calculate the converged charge density for the future band structure calculations.

### A. Band Structure

The 3D Bravais lattice is expressed using the real space translational vector:

(1) $$\vec{R}=n_{1}{\vec{a}}_{1}+n_{2}{\vec{a}}_{2}+n_{3}{\vec{a}}_{3}$$

where a_1_, a_2_, a_3_ are the real space lattice vectors.

The corresponding reciprocal space translational vector G can be expressed using:

(2) $${\vec{G}}_{m}=m_{1}{\vec{b}}_{1}+m_{2}{\vec{b}}_{2}+m_{3}{\vec{b}}_{3}$$

where b_1_, b_2_, b_3_ are the reciprocal space vectors.

(3) $${\vec{b}}_{1}=2\pi \frac{{\vec{a}}_{2}\;\times \;{\vec{a}}_{3}}{{\vec{a}}_{1}\;\cdot \;\left({\vec{a}}_{2}\;\times \;{\vec{a}}_{3}\right)}$$

(4) $${\vec{b}}_{2}=2\pi \frac{{\vec{a}}_{3}\;\times \;{\vec{a}}_{1}}{{\vec{a}}_{2}\;\cdot \;\left({\vec{a}}_{3}\;\times \;{\vec{a}}_{1}\right)}$$

(5) $${\vec{b}}_{3}=2\pi {\frac{{\vec{a}}_{1}\;\times \;{\vec{a}}_{2}}{{\vec{a}}_{3}\;\cdot \;\left({\vec{a}}_{1}\;\times \;{\vec{a}}_{2}\right)}}_{\;}$$

**Figure 1:**
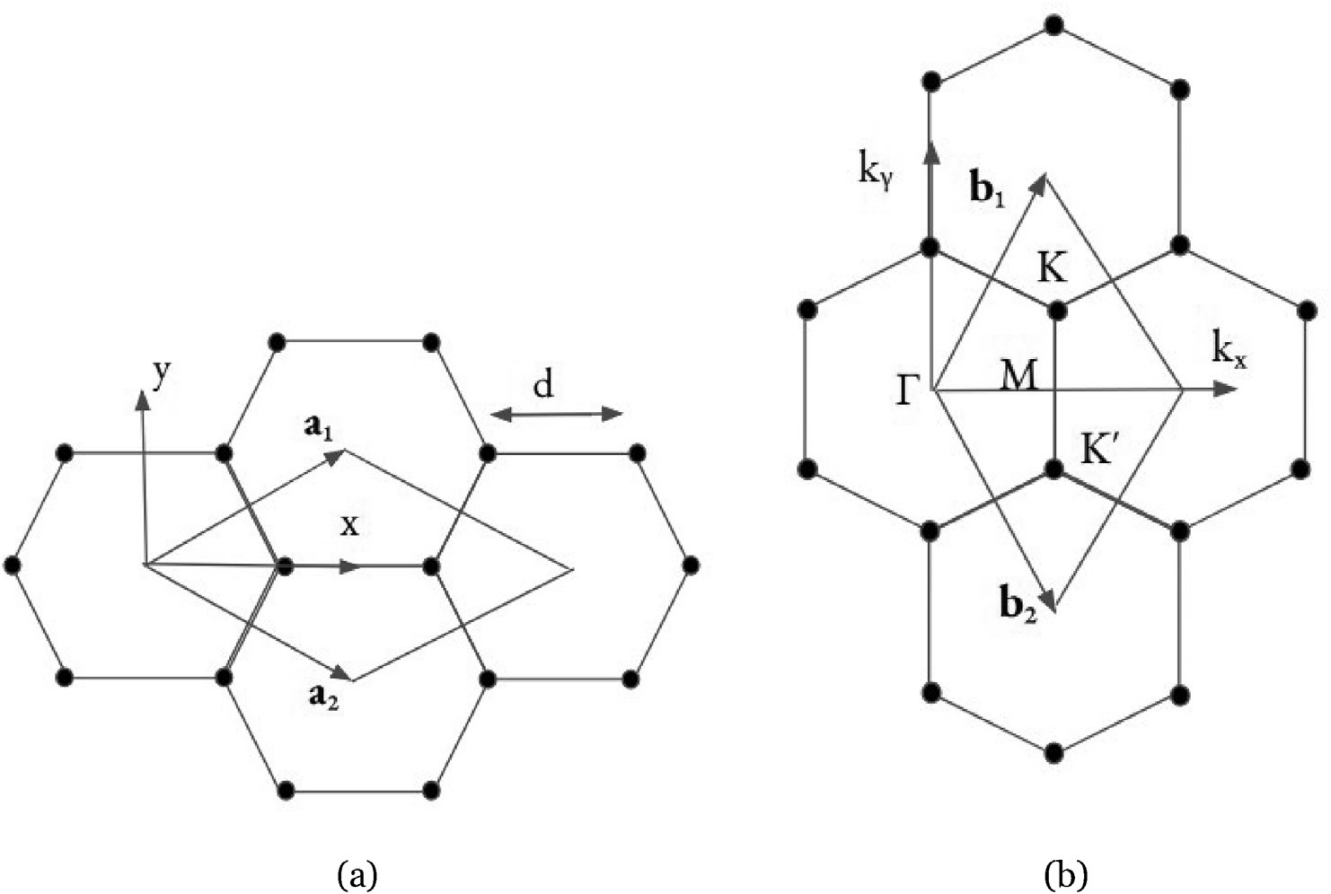
(a) Real space primitive cell of graphene defined by the unit vectors ${\vec{a}}_{1}$ and ${\vec{a}}_{2}$. (b) Reciprocal space primitive cell of graphene defined by the unit vectors ${\vec{b}}_{1}$ and ${\vec{b}}_{2}$. The high symmetry k points Γ(0, 0, 0), K (1/3, 2/3, 0), *K*′(2/3, 1/3, 0) and M (1/2, 0, 0), are also shown. This shows the first Brillouin zone of graphene, along with the high-symmetry k-points.

Figure 1(a) displays the Bravais lattice of graphene. The primitive cell is outlined with two of its basis vectors, ${\vec{a}}_{1}$ and ${\vec{a}}_{2}$, and contains two carbon atoms. The bond length, d, is 1.42 ˚A. Figure 1(b) displays the reciprocal space of graphene, representing the Brillouin Zone, where ${\vec{b}}_{1}$ and ${\vec{b}}_{2}$ outlines its basis vectors.

In order to perform band structure calculations, the first step was to compute the self-consistent total energy so all k-point sets could be calculated automatically with the.

Monkhorst-Pack scheme [34]. The resulted converged charge density was then used to perform the non-selfconsistent band structure calculations with the high symmetry k-points circuit Γ(0, 0, 0), M (1/2, 0, 0), K (1/3, 2/3, 0), Γ(0, 0, 0). We included four empty bands that simulated the conduction bands.

### B. Surface Calculations

We first have performed calculations of the atomic structure and band structure of pure graphene with the slab model and converged vacuum height. We used 2 × 2 supercell to calculate graphene doped with oxygen [35], boron [36], nitrogen [22], germanium [37], tin [38] and silicon [39] elements. In these calculations, the kinetic energy cutoff was chosen as the largest of the converged energy cutoffs of all the elements in the system, the k-point mesh was divided by two along the lateral directions as the lattice constant became doubled and the vacuum height was reconverged for each of the dopant systems. We then used these converged values to optimize our lattice constants and atom positions.

### C. Doping

**Figure 2:**
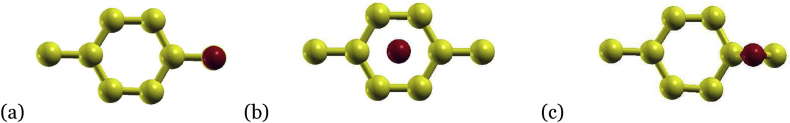
Three typical interstitial dopant sites (a) directly on top of a carbon atom (T-site) (b) the center of a hexagonal (H-site) (c) the midpoint of a C–C bond (B-site).

Graphene was interstitially doped using oxygen, boron and nitrogen. Each element was deposited on three typical doping sites, with either the doping element located directly on top of a carbon atom (T-site), at the center of a hexagonal (H-site) or the midpoint of a C–C bond (B-site); all three positions are shown in Figure 2.

Additionally, graphene was substitutionally doped using germanium, tin, and silicon elements.

### D. The Relationship Between Band Gap and the Color of Light

The relationship between band gap and the color of light is given by the following equation:

(6) $$E_{\lambda }=\left(hc\right)/\lambda$$

where *λ* represents wavelength and c is the speed of light. For a given frequency, the band gap is represented by *E*_*λ*_. In near-infrared light, the wavelength is 700–1000 nm and the corresponding band gap is 0.34–0.73 eV.

## 3 Results

### A. Convergence

Before relaxing the lattice vectors, we performed convergence calculations to obtain the most accurate energy cutoff, k-points and vacuum value for each element. In Table 2, the values obtained for each material are presented. In order to achieve high accuracy, we did compute the convergence of the total energy cutoff, k-points and vacuum convergence using the following criterion: a change in total energy of less than 0.0001 Hartree for two consecutive SCF cycles (see Table 1).

**Table 1:** The electron configurations and radius cutoff for each element utilized to generate the PAW pseudopotential [30].

Element Electron Configuration Radius Cutoff (Bohr)		
C	[*He*]2*s*^2^2*p*^2^	1.5
O	[*He*]2*s*^2^2*p*^4^	1.4
B	[*He*]2*s*^2^2*p*^1^	1.7
N	[*He*]2*s*^2^2*p*^3^	1.2
Ge	[*Ar*]3*d*^10^4*s*^2^4*p*^2^	2.3
Sn	[*Kr*]4*d*^10^5*s*^2^5*p*^2^	2.5
Si	[*Ne*]3*s*^2^3*p*^2^	1.9

**Table 2:** Converged kinetic energy cutoff, k-point mesh and vacuum height for 2 × 2 supercell.

Material	Energy Cutoff (Ha)	K-points	Vacuum (Bohr)
Pure Graphene	17	8 × 8 × 1	12
O doped graphene	20	8 × 8 × 1	24
B doped graphene	14	8 × 8 × 1	24
N doped graphene	25	8 × 8 × 1	24
Ge doped graphene	25	8 × 8 × 1	24
Sn doped graphene	25	8 × 8 × 1	24
Si doped graphene	25	8 × 8 × 1	24

### B. Relaxation

We used oxygen, boron, nitrogen, germanium, tin and silicon to dope pure graphene. For our elements using interstitial doping, oxygen, boron and nitrogen, we have selected three typical positions for each dopant material, so in total, we have optimized nine atomic structures. In addition to the interstitial doped materials, we have optimized three structures using substitutional doping for germanium, tin and silicon elements. Using our previously converged values, we optimized the lattice constant and atomic positions for all of the doped graphene materials with the criterion requiring 1.0 × 10^−5^ Hartree/Bohr. The optimized lattice constants are listed in Table 3.

**Table 3:** Optimized lattice constants for doped graphene materials.

Material	Lattice Constant Values (Bohr)
Pure Graphene	9.32 9.32 12
O doped (B-site)	9.41 9.41 25
O doped (H-site)	9.32 9.32 25
O doped (T-site)	9.37 9.37 25
B doped (B-site)	9.38 9.38 25
B doped (H-site)	9.37 9.37 25
B doped (T-site)	9.38 9.38 24
N doped (B-site)	9.34 9.34 25
N doped (H-site)	9.34 9.34 25
N doped (T-site)	9.39 9.39 25
Ge doped (sub)	10.12 10.12 25
Sn doped (sub)	10.55 10.55 25
Si doped (sub)	9.99 9.99 25

### C. Band Structure

In order to perform band structure calculations, we have first performed total energy calculations for all the materials. Using the three converged values, the kinetic energy cutoff, k-point mesh and vacuum height as well as the optimized lattice constants and atomic positions, we performed total energy calculations, resulting in obtaining the converged charge density. Using the converged charge density, we performed non-selfconsistent band structure calculations for the nine materials. In the final step, utilizing all the values we had obtained thus far, we plotted the band structure and calculated the band gap of each material. For the doped materials, we chose the smallest of the band gaps out of the three sites (B-site, H-site and T-site) that still fit in the ideal range. The results are presented in Table 4. The boron-doped graphene satisfied our required band gap of 0.3–0.7 eV.

**Table 4:** Calculated band gaps for graphene and graphene doped with various elements.

Material	Band Gap (eV)
Pure Graphene	0.0
O doped (B-site)	2.8
O doped (H-site)	0.0
O doped (T-site)	1.23
B doped (B-site)	0.69
B doped (H-site)	1.3
B doped (T-site)	0.68
N doped (B-site)	2.3
N doped (H-site)	0.0
N doped (T-site)	2.1
Ge doped (sub)	2.5
Sn doped (sub)	1.9
Si doped (sub)	1.82

**Figure 3:**
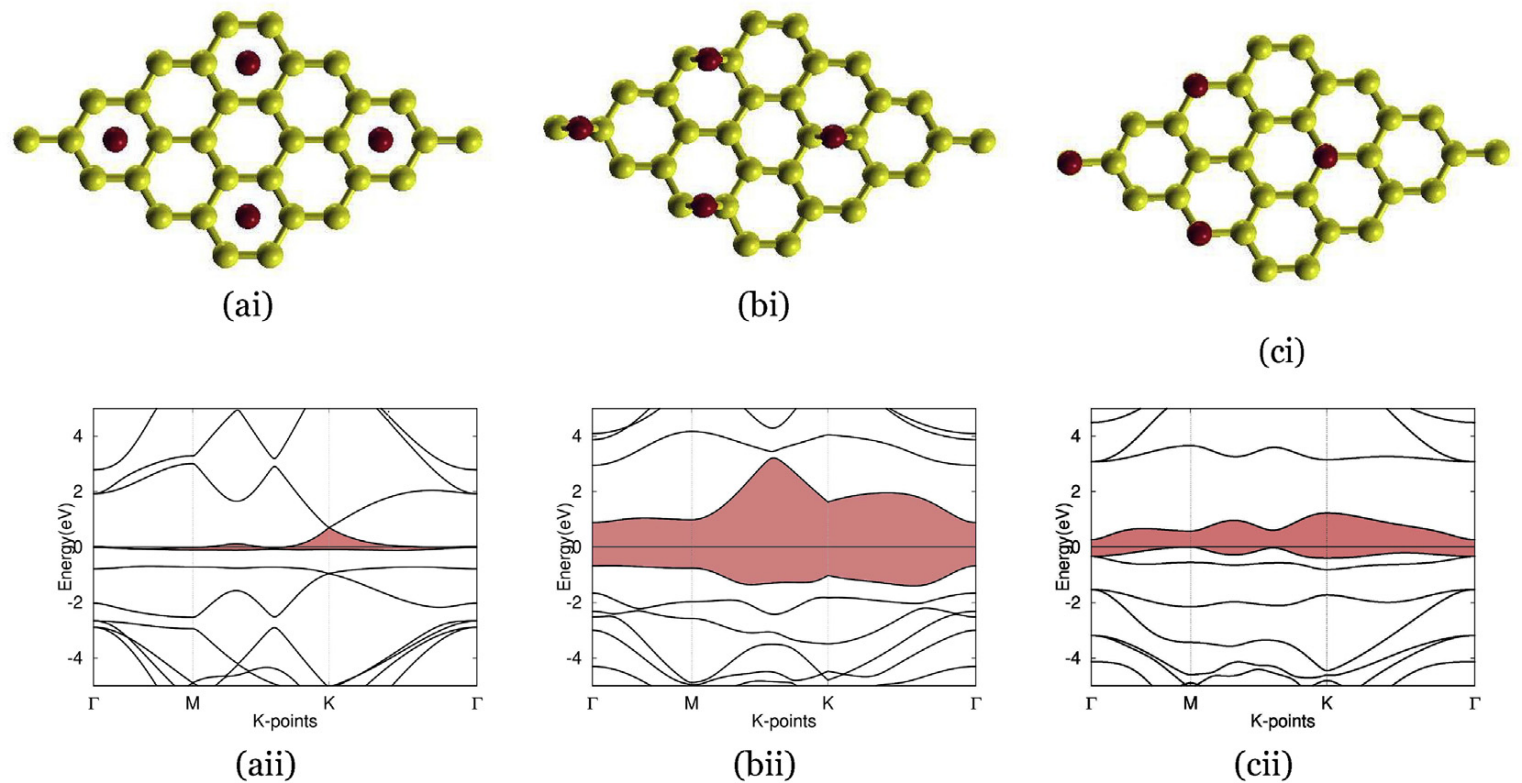
The three typical sites for interstitial doping using the element oxygen: (ai) and (aii) presents graphene doped with oxygen at H-site, with band structure on the bottom and atomic structure on the top, with an oxygen atom (red) deposited in the center of carbon rings. The band gap is highlighted in red, but because the valence and conduction band intersects, there is no band gap for this material. (bi) and (bii) present graphene doped with oxygen at B-site, with band structure on the bottom and atomic structure on the top, with an oxygen atom (red) deposited on the C–C bond. The band gap is highlighted in red, showing an indirect band gap of 2.8 eV. (ci) and (cii) Graphene doped with oxygen at T-site, with band structure on the bottom and atomic structure on the top, and with an oxygen atom (red) deposited on carbon atoms. The band gap is highlighted in red, showing an indirect band gap of 1.23 eV.

**Figure 4:**
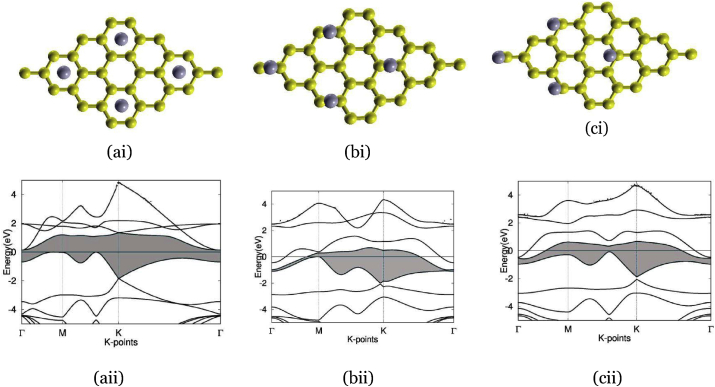
The three typical sites for interstitial doping using boron: (ai) and (aii) Graphene doped with boron at H-site, with band structure on the bottom and atomic structure on the top, and with a boron atom (gray) deposited in the center of carbon rings. The band gap is highlighted in blue, showing an indirect band gap of 1.3 eV. (bi) and (bii) Graphene doped with boron at B-site, with band structure on the bottom and atomic structure on the top, and with a boron atom (gray) deposited on the CB bond. The band gap is highlighted in blue, showing an indirect band gap of 0.69 eV. (ci) and (cii) Graphene doped with boron at T-site, with band structure on the bottom and atomic structure on the top, and with a boron atom (gray) deposited on carbon atoms. The band gap is highlighted in blue, showing an indirect band gap of 0.68 eV.

**Figure 5:**
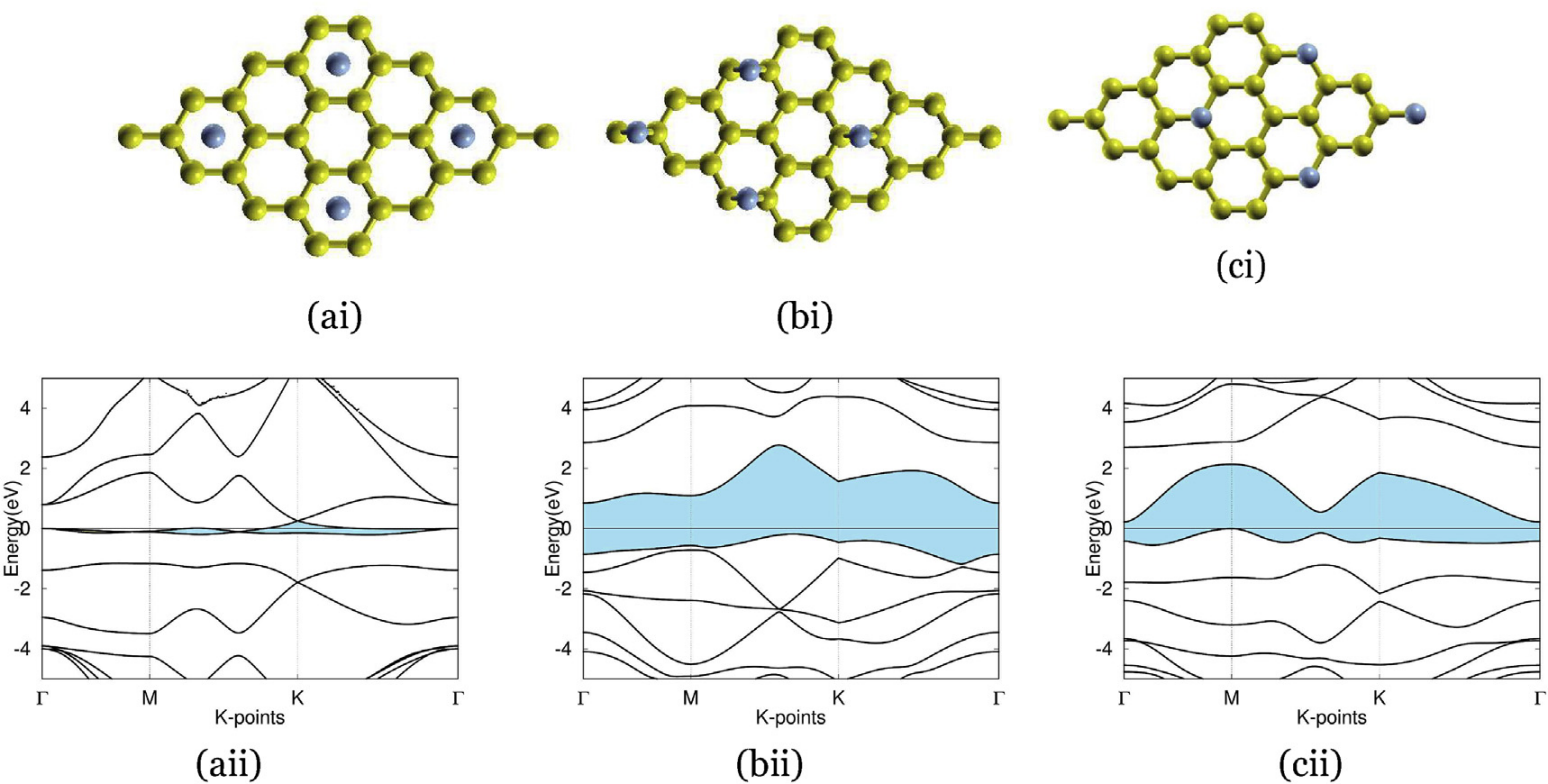
The three typical sites various sites for interstitial doping using the element nitrogen: (ai) and (aii) Graphene doped with nitrogen at H-site, with band structure on the bottom and atomic structure on the top, and with a nitrogen atom (blue) deposited in the center of carbon rings. The band gap is highlighted in blue, but because the valence and conduction band intersects, there is no band gap for this material. (bi) and (bii) Graphene doped with nitrogen at B-site, with band structure on the bottom and atomic structure on the top, and with a nitrogen atom (blue) deposited on CN bonds. The band gap is highlighted in blue, showing an indirect band gap of 2.3 eV. (ci) and (cii) Graphene doped with nitrogen at T-site, with band structure on the bottom and atomic structure on the top, and with a nitrogen atom (blue) deposited on carbon atoms. The band gap is highlighted in blue, showing an indirect band gap of 2.1 eV.

**Figure 6:**
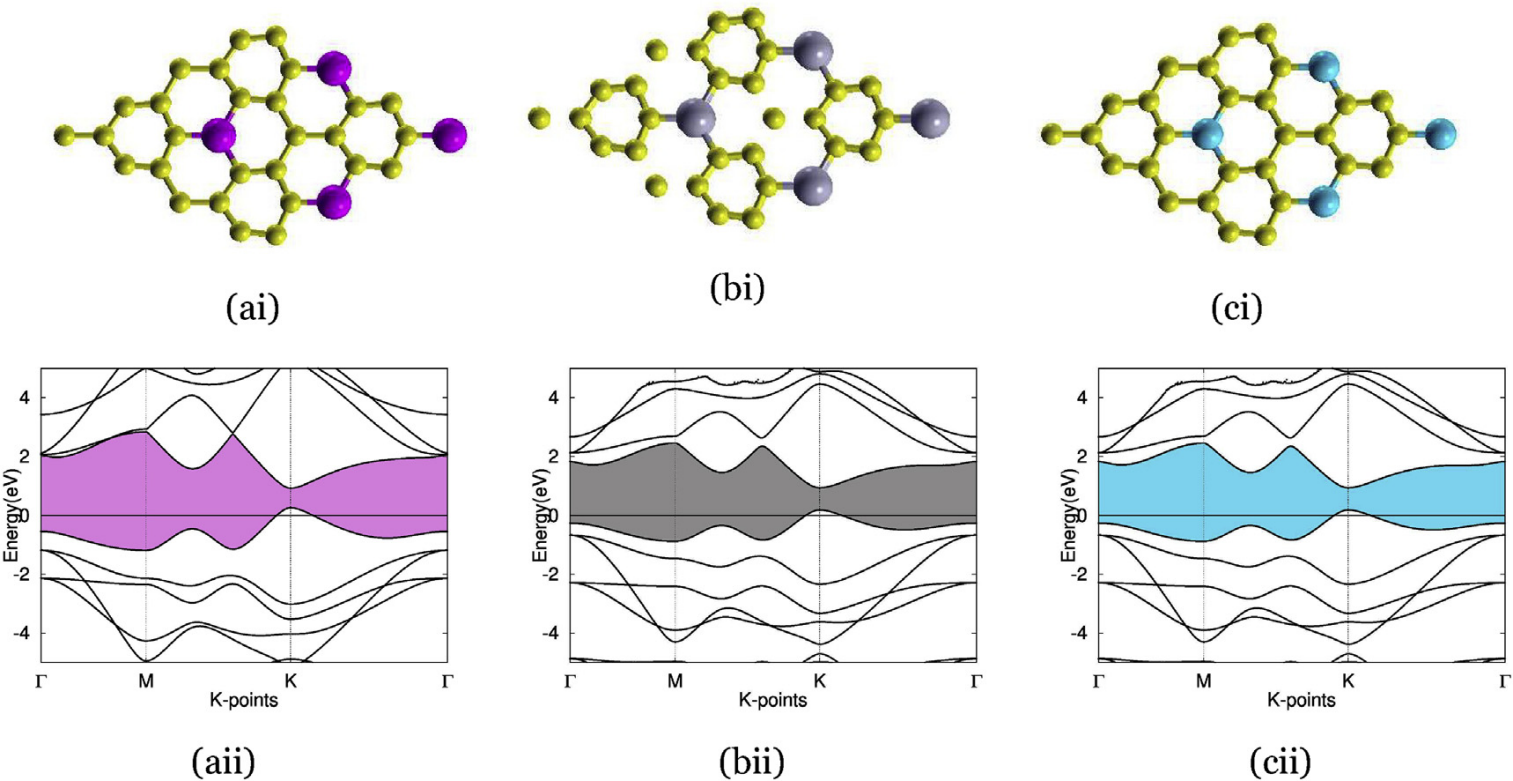
These three materials, germanium-doped graphene, tin-doped graphene, and silicon-doped graphene, all used substitutional doping. (ai) and (aii) Graphene doped with germanium, using substitutional doping. Bottom is band structure, top is atom structure, with a germanium atom (purple) replacing an atom in the graphene ring. The band structure depicts an indirect band gap of 2.5 eV. (bi) and (bii) Graphene doped with tin, using substitutional doping. Bottom is band structure, top is atom structure, with a tin atom (gray) replacing an atom in the graphene ring. The band structure depicts an indirect band gap of 1.9 eV. (ci) and (cii) Graphene doped with silicon, using substitutional doping. Bottom is band structure, top is atom structure, with a silicon atom (blue) replacing an atom in the graphene ring. The band structure depicts an indirect band gap of 1.82 eV.

In Figures 3, 4, 5, and 6, we present and elaborate on the optimized structure paired with the electronic band structure of our various dopants on a 2 by 2 supercell of graphene.

Figure 3 (ai) depicts the atomic structure of oxygen-doped graphene at the H-site. The bond length of C–O at the H-site increased to 0.243 nm compared to the standard C–C bond length in graphene of 0.142 nm and standard C–O bond length of 0.143 nm. Using interstitial doping, oxygen-doped graphene at the H-site resulted in an indirect band gap of 0.0 eV, as shown in (aii), which was not appropriate for our target range of 0.3–0.7 eV. Oxygen-doped graphene at the B-site is depicted by (bi), where the bond length of C–O at the B-site slightly increased to 0.146 nm from the standard C–C bond length in graphene and standard C–O bond length. By interstitially doping oxygen-doped graphene at the B-site, the result outputted an indirect band gap of 2.8 eV, which was too large with regard to our target range, shown by (bii). Lastly, oxygen-doped graphene at the T-site shown in (ci) depicted a bond length of 0.223 nm, nearly doubling relative to the typical bond length of C–C in graphene and standard C–O bond length. Using interstitial doping to create oxygen-doped graphene at the T-site resulted in a band gap of 1.23 eV, as shown in (cii).

Figure 4 (ai) represents the atomic structure of boron-doped graphene at the H-site. In comparison to the typical C–C bond length in graphene of 0.142 nm in graphene and standard C–B bond length of 0.156 nm, the bond length of C–B at the H-site increased to 0.229 nm. Through interstitial doping, boron-doped graphene at the H-site resulted in an indirect band gap of 1.3 eV, as shown (aii), which appeared to be too large for our optimal range of 0.3–0.7 eV. Boron-doped graphene at the B-site is shown in (bi), with the bond length of C–B increasing to 0.188 nm, as opposed to the typical C–C bond length in graphene of 0.142 nm and standard C–B bond length of 0.156 nm. Interstitially doping boron-doped graphene at the B-site, the outcome was an indirect band gap of 0.69 eV, as seen in (bii), which landed in the desired range of 0.3–0.7 eV, indicating the promising use of this material in photothermal therapy. Finally, boron-doped graphene at the T-site as shown as (ci), with an increased bond length of 0.233 nm, almost double the typical bond length of C–C in graphene and standard C–B bond length. Interstitial doping of boron-doped graphene at the T-site outputted a band gap of 0.68 eV, as shown in (cii), landing in the range of 0.3–0.7 eV-a potentially usable material.

Figure 5 (ai) shows nitrogen-doped graphenes atomic structure at the H-site, with the C–N bond length being 0.229 nm (vs C–C bond length in graphene of 0.142 nm and standard C–N bond length of 0.147 nm). The method of interstitial doping rendered an absence of a band gap in nitrogen-doped graphene at the H-site, as seen in (aii), not an appropriate size band gap for our purposes. (bi) depicts the atomic structure of nitrogen-doped graphene at the B-site, with a bond length of 0.147 nm, a small increase from the typical C–C bond length in graphene, however identical to the standard C–N bond length. Interstitially doped nitrogen-doped graphene at the B-site resulted in a band gap of 2.3 eV, shown in (bii), which was much too large to be of use. Lastly, nitrogen-doped graphene at the T-site, as shown in (ci), grew to a bond length of 0.147 nm, a slight increase from the typical C–C bond length in graphene and again, identical to the standard C–N bond length. The use of interstitial doping to create nitrogen-doped graphene at the T-site engineered a band gap of 2.1 eV, as seen in (cii), too large to be appropriate for our purposes.

Figure 6, (ai) shows germanium-doped graphenes atomic structure using substitutional doping, resulting in the bond length increase to 0.175 nm vs the standard C–C bond length in graphene of 0.142 nm and standard C–Ge bond length of 0.195 nm. Substitutionally doping graphene with germanium rendered a band gap of 2.5 eV, as shown in (aii), outside the range of 0.3–0.7 eV. The atomic structure of tin-doped graphene is seen in (bi), with a larger bond length of 0.189 nm, relative to the usual C–C bond length in graphene of 0.142 nm, however, less than the standard C–Sn bond length of 0.216 nm. The method of substitutional doping used for tin doped-graphene created a band gap of 1.9 eV, seen in (bii), much outside of the optimal range of 0.3–0.7 eV. (ci) represents the atomic structure of silicon-doped graphene and its bond length of 0.169 nm, an increase from the typical C–C bond length in graphene but decrease from the standard C–Si bond length of 0.185 nm. Substitutional doping resulted in a 1.8 eV band gap in silicon-doped graphene, seen in (cii)-again, too large for our purposes.

## 4 Discussion

Photothermal therapy needs a specific band light to excite a photosensitizer to a state where it is able to absorb vibrational energy in order to ablate the target tumor. Nanomaterials have been prime candidates in photothermal therapy as ablation agents to convert light to heat, due to their optimal optical absorption and electrical capabilities [40, 41]. In the past, out of the many nanomaterials tested in photothermal therapy, graphene has displayed satisfactory but not flawless results. Because of graphenes potential in the photothermal therapy treatment field, our research manipulated graphene as a base material to test with a multitude of dopants, with the hope of engineering a material with a specifically sized band gap. The dopants - oxygen, boron, nitrogen, tin, germanium, and silicon - were chosen due to their band gaps under 1.2 eV, an optimal size to open a band gap in the range of 0.3–0.7 eV. Our research used this design material as a potential select material in photothermal therapy.

In order to save experimental resources, theoretical calcations are required. Prior to an elevated concentration of attention and resources with respect to manufacturing materials for photothermal therapy, it was necessary to first optimize potential nanomaterials. Our research engineered twelve potential nanomaterials through doping, nine of which utilized interstitial doping and three of which utilized substitutional doping. Oxygen-, boron-, and nitrogen-doped graphene, each doped at the H-, B-, and T-site, constituted the nine interstitially doped materials, where a dopant atom was deposited on a graphene layer. Tin-, germanium-, and silicon-doped graphene constituted the three substitutionally doped materials, where a carbon atom was replaced with a dopant atom within the graphene structure. Boron doped graphene at the B- and T-site proved suitable as a nanomaterial to convert light to vibrational energy for tumor ablation, with band gaps of 0.69 eV and 0.68 eV respectively, fitting in the optimal range of 0.3–0.7 eV, in excellent agreement with previous calculations [42]. The ideal band gap of the two materials emphasized their potential to absorb enduring and concentrated near-infrared light for effective tumor ablation.

Since photothermal therapy operates through using near-infrared light lasers to ablate tumors on a patient, this method will most likely remain an option for malignancies near or on the surface of a cancer patient's body. However, if a more invasive tactic is needed due to tumors in the depths of a body cavity, photothermal therapy reaches its apex of effectiveness when used before or in conjunction with chemotherapy [43, 44] and surgery. By first eliminating the primary malignant cell mass, it is increasingly convenient for other forms of treatment to then attack the child tumors, formed from the parent.

Photothermal therapy is a relatively new and innovative treatment for many conditions, primarily cancer. The main obstacle hindering photothermal therapy from becoming a common and reliable treatment for cancer in the healthcare industry is the inefficient and inconsistent ability of materials used in the involved procedures. Using the knowledge of existing potential materials in the scientific sphere and theoretically engineering and testing compounded products, a choice nanomaterial to streamline the procedure of photothermal therapy will certainly be discovered. The breakthrough of photothermal therapy as an emerging treatment in the medical field will renew the simple and effective concept of utilizing heat to attack malignancies, bringing a non-invasive and less risky method into the realm of cancer treatment.

## 5 Conclusion

A wide range of materials have been introduced into the testing of photothermal therapy, depicting the high potential of this non-invasive and emerging cancer treatment. With the wonder material, graphene, as the base of our twelve nanomaterials, the potential for a successful ablation agent is large. Through our DFT calculations within the Abinit computational program suite, we found boron-doped graphene to be an unexpected but possible contender as an ablation agent in photothermal therapy, resulting in a band gap of 0.69 eV at B-site and 0.68 eV at the T-site. The band gap of boron-doped graphene matched the wavelength of near-infrared light, deeming it as an effective convertor of vibrational energy. As a possible ablation agent, boron-doped graphene holds many possibilities. Ablation agents in photothermal therapy require further evolvement to become multifunctional, as a drug carrier or contrast agent. In conclusion, our research serves to primarily theoretically optimize the chosen materials to be used in photothermal therapy, to save resources and money for the future of this treatment, prior to serious testing. Our research also serves the purpose opening a new avenue in the search of an ablation agent through the doping of graphene to create unique nanomaterials, which comprise a vast range of possibilities regarding the future of the medical field.

## Data availability

The raw/processed data required to reproduce these findings can be available via special request to the author.

## Declarations

### Author contribution statement

Faith Cheung: Conceived and designed the experiments; Performed the experiments; Analyzed and interpreted the data; Contributed reagents, materials, analysis tools or data; Wrote the paper.

### Funding statement

This research did not receive any specific grant from funding agencies in the public, commercial, or not-for-profit sectors.

### Competing interest statement

The authors declare no conflict of interest.

### Additional information

No additional information is available for this paper.
